# Research and Remedies

**Published:** 1913-03-22

**Authors:** 


### RESEARCH AND REMEDIES.

### The Treatment of Burns in Vienna.

In Lotheisen's clinique in Vienna the toxic factor is regarded as the most important one to combat when dealing with a case of severe burns. The routine practice, as given by Lieber, is to swab the burnt surface gently with benzine, and then to powder it thickly with novoiodine powder and to cover it with gauze. No attempt to render the skin absolutely aseptic is made; morphia and anæsthetics are avoided, as tending to promote or increase shock. When pain is very severe, anæsthesin powder is also used; this is both antiseptic and analgesic. The old dressing is removed in a bath when necessary, and the process repeated. Blisters are snipped before the novoiodine is applied. Cardiac stimulants are given freely, and a great point in the treatment is copious saline infusion, which is stated to have given admirable results. All wet dressings, ointments, and sedative drugs are avoided religiously.

### A Carcinoma Skin Reaction.

In 1910 three workers published in America a simple cutaneous reaction, which they held to be diagnostic in 90 per cent. or more cases of the presence or absence of malignant disease. The substance used for the test was a 20 per cent. suspension in normal saline of washed human blood corpuscles from a healthy individual. Subsequently it was shown that all human beings can be allotted to four groups on the basis of the behaviour of their red corpuscles; and it was only the fourth group whose cells were of use for the purposes of this particular test. Lisser and Bloomfield have used corpuscles from persons belonging to Group IV. only, and their results are published in the *Johns Hopkins Hospital Bulletin*. In 62 cases of verified malignant disease, two-thirds gave a positive and one-third a negative reaction. In 94 cases of disease other than malignant there were 8 positive and 86 negative reactions. On these figures they remark that a negative reaction adds little or no weight to the evidence against cancer, whereas a positive one is very fairly strongly in favour of that diagnosis. The non-malignant cases that gave positive reactions were: infective arthritis, osteoarthritis, gall stones, chronic appendicitis, tuberculosis of breast, endocervicitis, burn of arm in early toxic stage, and purpura. As a prognostic help the test appears to have little value; for in a case of cancer of the breast the reaction was negative immediately after a radical operation, yet recurrence occurred within a month; a week after the recurrence the reaction was once more positive.

### Hæmorrhagic Disease of the New-born.

Once in a while the practitioner who does much obstetric work will come across a case in which an infant of a few days old will start to bleed for no apparent reason and within a few days will slowly bleed to death in spite of all efforts to save it. As a rule the umbilicus is the principal source of this hæmorrhage, but the intestinal tract is also liable to be affected, and hæmatemesis or melæna thus results. The operation of circumcision is also sometimes the provoking agent in starting the mischief. Of late a great deal of work has been done with the object of improving the chances of these distressing cases; animal serum and human blood serum injected subcutaneously have been favourably reported upon by several authorities. Later still, human blood itself was used in the same way with considerable success; and then it was suggested to transfuse blood directly into one of the infant's veins. In the *Archives of Pediatrics* Dr. Beth Vincent narrates his experiences with the latter method in eleven very serious cases of this disease. In eight cases success was immediate and permanent; and in the other three the causes of failure were independent of and beyond the control of the remedy. In every case bleeding ceased at once as soon as the transfusion took place, and in the successful cases the patients were transformed at once from desperately sick to healthy children. The source of supply was the father's radial artery in most of the cases, and the blood was led directly through a glass cannula into the infant's jugular vein. The technique does not seem to be excessively difficult, though necessarily the aseptic precautions must be beyond criticism. The method certainly promises well, and is worth trial by anyone who encounters one of these intractable and disastrous cases.

### Chloretone for Chorea.

Trichlor - tertiary - butyl - alcohol, more familiar under the name of chloretone, is well known for its general and local sedative properties. It has been especially used for sea-sickness and for local application to hæmorrhoids. In the *Archives of the Middlesex Hospital* Dr. Wynter strongly recommends it for chorea, having prescribed it for ninety consecutive cases with satisfactory results. He has usually given five-grain doses every four hours; this quantity he doubles when dealing with an adolescent. The drug is ordered in an emulsion of paraffin or of cod-liver oil; and, if necessary, it can be given in an enema, solution being effected by the addition of spirit or glycerin. This dosage is continued for forty-eight hours, after which a longer interval is usually sufficient, as the movements and restlessness diminish. Should drowsiness supervene, the chloretone is intermitted for a day or two until this effect passes off. The drug is useful for the chorea of pregnancy as well as for that of childhood. Dr. Wynter does not find that there is much shortening of the average duration of the stay in hospital of the patients thus treated; but he thinks the period of active symptoms is materially shortened, though the total duration of convalescence may not be much affected. It is noteworthy also that in two cases exfoliation of the skin occurred, simulating the desquamation which follows scarlet fever.

